# Obesity-Driven Gut Microbiota Inflammatory Pathways to Metabolic Syndrome

**DOI:** 10.3389/fphys.2015.00341

**Published:** 2015-11-19

**Authors:** Luiz H. A. Cavalcante-Silva, José G. F. M. Galvão, Juliane Santos de França da Silva, José M. de Sales-Neto, Sandra Rodrigues-Mascarenhas

**Affiliations:** ^1^Programa Multicêntrico de Pós-graduação em Ciências Fisiológicas, Laboratório de Imunofarmacologia, Centro de Biotecnologia, Universidade Federal da ParaíbaJoão Pessoa, Brasil; ^2^Programa de Pós-Graduação em Produtos Naturais e Sintéticos Bioativos, Laboratório de Imunofarmacologia, Centro de Ciências da Saúde, Universidade Federal da ParaíbaJoão Pessoa, Brasil; ^3^Programa de Pós-Graduação em Biotecnologia, Laboratório de Imunofarmacologia, Centro de Biotecnologia, Universidade Federal da ParaíbaJoão Pessoa, Brasil

**Keywords:** adipose tissue, cytokines, gut microbiota, immune system, toll-like receptors

## Abstract

The intimate interplay between immune system, metabolism, and gut microbiota plays an important role in controlling metabolic homeostasis and possible obesity development. Obesity involves impairment of immune response affecting both innate and adaptive immunity. The main factors involved in the relationship of obesity with inflammation have not been completely elucidated. On the other hand, gut microbiota, via innate immune receptors, has emerged as one of the key factors regulating events triggering acute inflammation associated with obesity and metabolic syndrome. Inflammatory disorders lead to several signaling transduction pathways activation, inflammatory cytokine, chemokine production and cell migration, which in turn cause metabolic dysfunction. Inflamed adipose tissue, with increased macrophages infiltration, is associated with impaired preadipocyte development and differentiation to mature adipose cells, leading to ectopic lipid accumulation and insulin resistance. This review focuses on the relationship between obesity and inflammation, which is essential to understand the pathological mechanisms governing metabolic syndrome.

## Gut microbiota role in obesity

Obesity has increased alarmingly worldwide, promoting mortality and morbidity (Mitchell and Shaw, 2015). Overweight and obesity are commonly associated with accumulated abdominal visceral fat and can be related to psycho-sociological behavioral disorders (Burdette and Hillb, 2008; Jauch-Chara and Oltmanns, 2014). Fat gain and adipose tissue inflammation, resulted from excessive caloric intake and reduced energy expenditure, lead to positive energy balance and can contribute to metabolic syndrome (Trayhurn, 2005; Emanuela et al., 2012; DeMarco et al., 2014). Besides, chronic stress and gut microbiota deregulation can affect obesity development (McGill, 2014).

Human microbiota, made up of bacteria, archaeas, viruses and unicellular eukaryotes, represents more than 10^14^ microbial cells/humam, which live peacefully in our body (Sekirov et al., 2010). These microbes are found in our skin, genitourinary, respiratory and gastrointestinal tracts. Gut microbiota represents over than 7 × 10^13^ microbial cells/human, but its composition can be altered throughout life, including changes in gene expression (Walsh et al., 2014).

There are over 50 bacterial phyla, but the human gut microbiota is dominated mostly by the Bacteroidetes and the Firmicutes (Schloss and Handelsman, 2004; Sekirov et al., 2010). Gut specific microbial phyla, species and strains of humans and other animals are related to gene expression alterations observed in obesity (Ley et al., 2005; Turnbaugh et al., 2008; Fujimura et al., 2010; Clarke et al., 2012; Cotillard et al., 2013; de Theije et al., 2014). It has been demonstrated that obesity is associated with reduced bacterial diversity and modified representation of bacterial genes and metabolic pathways (Turnbaugh et al., 2009). Furthermore, Turnbaugh et al. (2006) provide evidences that gut microbiota in obese mice have an increased ability for energy harvest from the diet. In this work, colonization of germ-free mice with caecal microbiota harvested from obese donors results in a significant total body fat gain.

Probiotics (e.g., many bacterial strains of the *Lactobacillus* and *Bifidobacterium* genera), when administered in adequate amounts, induces health-beneficial effects, representing a novel anti-obesity mechanism (Raoult, 2009; Aronsson et al., 2010; Kadook et al., 2010). Studies demonstrated that *Lactobacillus* treatment reduces fat accumulation and pro-inflammatory cytokines in adipose tissue (Park et al., 2013; Yoo et al., 2013; Miyoshi et al., 2014; Ukibe et al., 2015). *Lactobacillus* strain (*L. plantarum*) anti-inflammatory effect was also observed in intestinal inflammation rat model, mostly by NF-κB (nuclear factor kappa-light-chain-enhancer of activated B cells) inhibition (Štofilová et al., 2015). Similar results were also observed in endotoxin- and metabolic-related inflammatory process in rats (Vilahur et al., 2015). However, in diabetic and non-diabetic individuals, oral supplementation with another *Lactobacillus* strain (i.e., *L. acidophilus*) did not affect systemic inflammatory response (Andreasen et al., 2010). These opposite results could be related to differences in *Lactobacillus* strains or even to different experimental models.

*Lactobacillus* effect on fat storage may involve upregulation of circulating lipoprotein lipase inhibitor, angiopoietin-like 4 protein (ANGPTL4), which controls triglyceride deposition into adipocytes (Aronsson et al., 2010). In addition, probiotics treatment can modulate gut flora composition, which in turn enhance metabolic functions to prevent overweight and obesity (Park et al., 2013; Yadav et al., 2013). Moreover, obese mice antibiotics treatment is also capable to reduce adiposity and adipose tissue inflammation, which reinforce the benefits of gut microbiota regulation (Tremaroli and Bäckhed, 2012).

Gastrointestinal microbiota also interferes with carbohydrate, lipid and amino acid metabolism (Hooper et al., 2002), complementing our own human metabolic apparatus (Bäckhed et al., 2004, 2007; Cani and Delzenne, 2009; Rabot et al., 2010). Thus, human gut microbiota can regulate many metabolic pathways, including bile acids biotransformation, which involves deconjugation, dehydroxylation, and reconjugation reactions (Ridlon et al., 2014). Gut microbiota components, such as bacterial bile salt hydrolases and bacterial 7α-dehydroxylase, can control these reactions and, thus, maintain bile acids pool size and composition (Ridlon et al., 2006). It has been demonstrated that bile acids have both direct antimicrobial effects on gut microbes and indirect effects through FXR (farnesoid X receptor)-induced antimicrobial peptides (Inagaki et al., 2006). This antimicrobial effect promoted by bile acids prevent mucosal injury in the small intestine and other injuries caused by excessive bacterial proliferation (Hofmann and Eckmann, 2006; Merritt and Donaldson, 2009). It was also described that reduced bile acid levels in the gut are associated with bacterial overgrowth and inflammation. However, some bacteria, such as *Alistipes, Bilophila*, and *Bacteroides*, are bile acids tolerant, which could lead to other symbiotic microbes suppression (David et al., 2014).

Bile acids can also regulate adiposity and glucose homeostasis. Studies demonstrated that nuclear receptor FXR deficiency leads to mass adipose tissue reduced levels (Cariou et al., 2006; Prawitt et al., 2011). On the other hand, FXR absence has different effects on glucose homeostasis in lean and obese mice. FXR^−∕−^ lean mice presents impaired glucose tolerance and insulin resistance (Cariou et al., 2006; Ma et al., 2006), while obese mice (murine models of genetic and diet-induced obesity) presents glucose homeostasis improvement (Prawitt et al., 2011). This difference can be explained by bile acids action in other receptors, such as TGR5 (also known as G protein bile acid receptor-1), since Thomas et al. (2009) showed that TGR5 activation results in the maintenance of glucose homeostasis and insulin sensitivity in obese mice.

Furthermore, gut microbiota plays a physiological role in host immune system development [e.g., gut-associated lymphoid tissue (GALT) development] (Bäckhed et al., 2005; Willing et al., 2010; Guinane and Cotter, 2013) and immune tolerance modulation (Bailey et al., 2005; Vael and Desager, 2009; Martin et al., 2010; Belkaid and Hand, 2014). In addition, gut microbiota modulates other important intestinal functions such as angiogenesis and epithelium function (Hooper et al., 2001). Epithelial (e.g., enterocytes and goblet cells) and endocrine cells provide an interplay between the host and its own gut microbiota via receptors such as toll-like receptors (TLRs; Lotz et al., 2003; Kelly et al., 2004; Hornef and Bogdan, 2005; Shibolet and Podolsky, 2007; Wells et al., 2011; Pott and Hornef, 2012). After TLR activation, pro-inflammatory molecules can be produced in the gut microbiota and impair host metabolism, which in turn can further cause adipose inflammation and obesity (Sanz and Moya-Pérez, 2014).

Additionally, gut homeostasis is related to other innate immune receptors, such as nucleotide-binding oligomerization domain (NOD) like receptors (NLR; Zambetti and Mortellaro, 2014). This family of cytosolic receptors includes NOD1/2 and NLRPs (NLR family, pyrin-domain-containing proteins). After activation, NLRP forms signaling complexes called inflammasomes, which generate active forms of the inflammatory cytokine IL-1β and IL-18. Some different inflammasome subtypes have been described such as NLRP1, NLRP3, NLRP6, NLRC4, AIM2 (Latz et al., 2013). Studies have demonstrated that NLRC4 inflammasome is involved in mucosal protection against infections (Sellin et al., 2014; Nordlander et al., 2014), while NLRP6 and NLRP3 are associated with gut microbiota homeostasis (Elinav et al., 2011; Hirota et al., 2011; Wlodarska et al., 2014). Inflammasomes and gut homeostasis interaction is substantially detailed by Sellin et al. (2015) and Zambetti and Mortellaro (2014).

## Interplay between inflammation and obesity

Inflammation is a tightly controlled physiological process that is orchestrated by immune system (Ashley et al., 2012), but is also regulated by other systems, such as endocrine (de Vasconcelos et al., 2011; Leite et al., 2015; Ren et al., 2015) and nervous system (Martelli et al., 2014; Bassi et al., 2015). Despite the protective body response represented by inflammation, deregulated, or excessive immune response can lead to several chronic diseases such as hypertension (Mirhafez et al., 2014), Alzheimer (Takeda et al., 2014), and obesity (Khan et al., 2014). The classical acute inflammatory process includes five cardinal signals: redness, heat, swelling, pain, and, eventually, loss of function (Medzhitov, 2010). These macroscopic signals are reflex of vascular (e.g., vascular permeability) and cellular (e.g., leukocytes migration) alterations during inflammation (Medzhitov, 2008). However, inflammatory response in obesity has some particular features (Gregor and Hotamisligil, 2011). Obesity involves immune response impairment affecting both innate and adaptive immunity. However, the mechanisms involved in the relationship between obesity and inflammation have not been completely elucidated (Sanz and Moya-Pérez, 2014).

Obesity is related to inflamed adipose tissue and increased local cell infiltration (Gregor and Hotamisligil, 2011). Different cell types contribute to adipose tissue inflammation, among these cells monocytes/macrophages play a critical role in this process (Cinti et al., 2005; Subramanian and Ferrante, 2009; Ferrante, 2013). Yoshimura et al. (2015) demonstrated that obese young adults have increased number of leukocytes, mostly monocytes, when compared with non-obese individuals. Also, elevated monocytes level is positively correlated with visceral subcutaneous fat as well as with body fat mass. Peripheral blood of obese women presents an elevated inflammatory monocytes amount (Ziegler-Heitbrock, 2007; Krinninger et al., 2014). In addition, Poitou et al. (2011) also demonstrated that inflammatory monocytes are increased in obese individuals and fat body loss is associated with significant decrease of these cells.

Once within tissues, monocytes differentiate in M1 or M2 polarized macrophages (Dalmas et al., 2011). The first type is classified in pro-inflammatory cell which expresses inducible nitric oxide synthase and pro-inflammatory cytokines (e.g., IL-6 and TNF-α), while M2 macrophages express arginase (Arg1) and the anti-inflammatory cytokine IL-10. In lean individuals, M2 macrophage predominates in adipose tissue unlike in obese individuals; wherein M1 macrophages are mostly present (Kraakman et al., 2014). Macrophages of high-fat diet fed mice display autophagy impairment, a cytoprotective response to different stimulus, which leads to M1 polarization (Liu et al., 2015).

In obese individuals, monocytes up-regulate chemokine receptor type 2 (CCR2) and thus they migrate toward adipose tissue. Despite the natural ligand of this receptor, the chemokine CCL2 (also as known as MCP-1), plays an important role in adipose tissue macrophage recruitment (Kanda et al., 2006), other studies demonstrated that CCL2 is not critical for macrophage infiltration into adipose tissue (Inouye et al., 2007; Kirk et al., 2008). These findings can be related to macrophage recruitment toward adipose tissue by other chemokine, such as CXCL12 and CXCL14, as demonstrated by Kim et al. (2014) and Nara et al. (2007), respectively. Furthermore, the chemokine CCL5 (also as known as RANTES) and its receptors CCR5 are also important in this macrophage migration process (Keophiphath et al., 2010; Kitade et al., 2012).

Additionally, CCR2 modulates other parameters than macrophage recruitments. High-fat diet fed mice with genetic CCR2 deficiency present food intake reduction and lower obesity development (Weisberg et al., 2006). In addition, obese CCR2^−∕−^ mice have an increased adipose tissue eosinophil number and high levels of IL-4 and IL-13, cytokines which lead to M2 macrophage polarization (Bolus et al., 2015).

Not only migration, but also macrophage proliferation contributes to adipose tissue inflammation. Amano et al. (2014) showed that obese mice increased macrophage proliferation, especially in visceral adipose tissue. Moreover, they showed that CCL2 stimulates adipose tissue macrophage proliferation.

Adipose tissue macrophages are source of inflammatory cytokines in obese individuals. Between these cytokines, IL-6 displays pleiotropic role in metabolism and obesity. Sárvári et al. (2015) demonstrated that macrophages engulf portions of adipocytes *in vitro* leading to NF-κB activation and IL-6 secretion. In addition, Kraakman et al. (2015) related pro-inflammatory action to IL-6 *trans*-signaling, a process where IL-6 binds a soluble receptor to trigger inflammation. In this work, they demonstrated that this IL-6 signaling induces macrophage recruitment to adipose tissue.

IL-6 can also induce C reactive protein (CRP) liver production, which is associated to complement activation, phagocytosis and cytokines production (Deban et al., 2009; Du Clos, 2013). In obese individuals, CRP is elevated, demonstrating a state of active immune response and inflammation in these subjects (Shaharyar et al., 2015; Yoshimura et al., 2015). On the other hand, Ma et al. (2015), using a different model, showed that sustained IL-6 gene expression in obese mice reduces body weight loss, fatty liver and insulin resistance. Additionally, it was evidenced that IL-6 supports M2 polarization, an anti-inflammatory cell, by sensitizing macrophages to IL-4 (Mauer et al., 2014). Despite its variable effects, these findings demonstrate IL-6 critical role of in obese individuals.

Although macrophages infiltration is considered a hallmark of adipose tissue inflammation, other cells of the immune system display a fundamental role (Sell et al., 2012). In fact, some studies demonstrated that neutrophil migration into adipose tissue, as well as in classical acute inflammation, occurs after 3 days of high-fat diet in mice (Elgazar-Carmon et al., 2008; Talukdar et al., 2012). In addition, Xu et al. (2015) demonstrated an increased peripheral blood neutrophil percentage in obese young male.

Several types of lymphocytes interact with other cells in adipose tissue environment to enhance or decrease inflammatory response. Interactions between macrophages and CD4^+^ T cell via MHC class II is required for adipose tissue inflammation and for obesity-induced insulin resistance (Cho et al., 2014). CD4^+^ T cell could polarize to different subtypes of lymphocytes, namely Th1, Th2, Th17, regulatory T (Treg) cells, and other types of cells (Luckheeram et al., 2012). Despite all these subtypes of cells are related to obesity and metabolic syndrome, pro-inflammatory Th1 and Th17 predominate over Treg and Th2 during adipose tissue inflammation (Sell et al., 2012; McLaughlin et al., 2014).

High-fat diet fed mice present Th1 polarized and IFN-γ production predominance, which occurs after macrophage recruitment (Strissel et al., 2010). IFN-γ expression displays a regulatory role in adipose tissue inflammation, since its absence reduces TNF-α and CCL-2 mRNA expression and macrophage adipose tissue accumulation (Rocha et al., 2008). Interestingly, T-box transcription factor (T-bet) absence, a key factor to development of Th1 cell, leads to obesity possibly by IL-6 up-regulation (Kim et al., 2013). In Table 1, we summarize other types of lymphoid cells involved in obesity-related inflammation.

**Table 1:** **Role of lymphoid origin cells in obesity-related inflammation**.

**Lymphoid subsets cells**	**Role in obesity**
Th17	Increased in obese individuals (Winer et al., 2009). IL-17A, a Th17 key cytokine, up-regulates IL-6, IL-8, and PGE2 levels in adipocytes (Shin et al., 2009).
Th22	Increased in obese individuals (Zhao et al., 2014). Unclear role.
NK cell	Contributes to M1 macrophage polarization (Wensveen et al., 2015).
iNKT	Induce M2 macrophage polarization and control Treg proliferation (Lynch et al., 2015).
ILC2s (group 2 innate lymphoid cells)	Control obesity development by inducing caloric expenditure (Brestoff et al., 2014).

Immune cells need to sense foreign structures to develop an immunological response. Particularly, innate immune cells use pattern recognition receptors (PRR) to recognize specific pathogen or damaged molecules (Janeway and Medzhitov, 2002). Between these receptors, toll-like receptors are structurally and functionally well-defined (Kawai and Akira, 2010), and are related to obesity.

## Toll-like receptors (TLR) and obesity

TLRs (toll-like receptors) can recognize pathogen-associated molecular patterns (PAMPs) of microorganisms, which are not conserved in eukaryotes. This recognition triggers immune system activation, setting up innate immune response (Kawai and Akira, 2010). These receptors were initially identified in the fruit fly *Drosophila melanogaster*, first being associated with its embryonic development. Later on, its role on pathogens detection and immune response was described (Lemaitre et al., 1996; Williams et al., 1997). Janeway and his collaborators identified the first toll homolog in humans, the TLR4 (Medzhitov et al., 1997). In mammals, there are 12 members from TLRs family, but only TLR1-TLR10 function is known (Akira et al., 2006).

TLRs location is important to grant the access to the ligand. The majority of plasma membrane TLRs recognizes microbial membranes components, such as proteins, lipoproteins and lipids; while intracellular TLRs are able to recognize nucleic acids of microorganism (Werling and Jungi, 2003). TLRs can recognize a broad variety of PAMPs derived from many classes of microorganisms such as parasites, fungi, viruses and bacteria (Medzhitov, 2007). These PAMPs include many molecules including β-glucan, found on fungus, both viral RNA and DNA, and also a huge quantity of elements derived from bacteria (e.g., lipopeptides, peptidoglycan, lipoteichoic acid, and lipopolysaccharide (LPS; Aderem and Ulevitch, 2000).

Despite the fact that TLRs recognize a variety of PAMPs, each TLR can only recognize a limited group of patterns and, therefore, has a determined specificity for their ligands (Beutler, 2003). TLR4 is the LPS receptor (Poltorak et al., 1998a,b). TLR2 was found to recognize bacterial peptidoglycan and lipopeptide (Takeuchi et al., 1999). TLR5 is able to recognize flagellin, a protein derived from bacterial flagella (Hayashi et al., 2001). TRL3 is associated to the identification of double-stranded RNA molecules (Alexopoulou et al., 2001). TLR7 can recognize RNA molecules, especially small interfering RNAs (Hornung et al., 2005). TLR8 is similar to TLR7 and recognize viral ssRNA. Finally, TLR9 is associated with the recognition of non-methylated bacterial DNA (Hemmi et al., 2000). Together, all these receptors are able to recognize a broad variety of microorganisms and promote activation of the NF-κB, which is responsible for synthesis of inflammatory mediators (Lee et al., 2012).

TLRs are specially expressed in hematopoietic cells, including immune system cells. However, its expression was already confirmed in other kind of cells such as adipocytes (Kanczkowski et al., 2008). Therefore, these receptors can act promoting interplay between the innate immune system and metabolism (Fresno et al., 2011). Studies conducted on the role of TLRs on adipose tissue suggest that all subtypes of TLRs can be found in this tissue. (Hwa et al., 2006; Pietsch et al., 2006; Poulain-Godefroy and Froguel, 2007; Vitseva et al., 2008). Nevertheless, initially only TLR2 and TLR4 were functional in human adipocytes (Bès-Houtmann et al., 2007), but lately TLR5 activation was evidenced (Pekkala et al., 2015). It was described that TLR2, TLR4, or TLR5 deficiency have a major role on obesity development (Fresno et al., 2011).

It was described that TLR2 activation can be triggered by saturated fatty acids (SFAs; Lee et al., 2001, 2003). During endotoxemia, TLR2 is also activated by bacterial peptidoglycan from the intestines (Cani et al., 2008). Moreover, TLR2 absence decreases expression of inflammatory mediators and macrophages infiltration in white adipose tissue (WAT). Also, other studies demonstrated that TLR2 reduced levels protects against obesity and inflammation (Himes and Smith, 2010; Davis et al., 2011). Together, these data reveal a certain importance regarding TLR2 role in obesity. In addition, the role played by TLR5 in obesity is not well-established. It was recently found that TLR5 signaling in adipose tissue could corroborate to obesity, inflammation and metabolic alterations. Additionally, it was reported that TLR5 activation leads to ERK1/2 (extracellular signal-regulated kinase) phosphorylation and adipocytes insulin signaling inhibition (Pekkala et al., 2015).

Both obesity and metabolic syndrome are characterized by inflammatory responses, triggered by adipose tissue disruption mediated TLR signaling (Pekkala et al., 2015). After activation, individual TLRs recruit TIR (Toll/IL-1 receptor) domain-containing adaptors members such as MyD88 (Myeloid differentiation primary response gene 88), TRIF (TIR-domain-containing adapter-inducing interferon-β), TIRAP/MAL (Toll-interleukin 1 receptor domain containing adaptor protein/ MyD88 adapter-like) or TRAM (TRIF-related adaptor molecule). However, MyD88 is used by all TLRs to activate NF-κB and MAPKs (mitogen-activated protein kinases) for the induction of inflammatory cytokine genes (Kawasaki and Kawai, 2014).

## TLR4 and cell signaling proteins: Targets to obesity and its complications

Obese patients express high levels of TLR4 (Reyna et al., 2008). TLR4 activation, which occurs in obesity, can be activated by gut microbial patterns, such as LPS, to promote inflammatory mediators production (Kim et al, 2012). In addition, TLR4 can also mediate the pro-inflammatory effect of SFAs, often found at high levels in plasma of obese individuals (Lee et al., 2001; Shi et al., 2006; Dasu and Jialal, 2010). Many studies demonstrated that decreased TLR4 expression protects from obesity development, adipose tissue inflammation and insulin resistance (Shi et al., 2006; Suganami et al., 2007; Tsukumo et al., 2007; Davis et al., 2008; de Mello et al., 2008). A similar effect was observed using anti-TLR4 antibodies (Milanski et al., 2009). In TLR4 deficient mice, adipose tissue inflammation reduction could be explained by M2 macrophage polarization (Orr et al., 2012).

Studies suggest that obesity TLR4 signaling essentially depends on MyD88 expression and up-regulated NF-κB activity, with IL-6 and TNF-α pro-inflammatory cytokines increased expression (Fresno et al., 2011). Despite this classical signaling pathway, new insights about TLR4 signaling are emerging. In fact, Luo et al. (2014) demonstrated that small GTPase Rab8a and phosphatidylinositol 3-kinase γ (PI3Kγ) act as regulators of cytokines production, decreasing pro-inflammatory cytokines and increasing anti-inflammatory cytokines. These effects are mediated by Akt/mTOR signaling. The protein kinase mTOR restrains the pro-inflammatory cytokines production by NF-κB inhibition, while the anti-inflammatory cytokine (i.e., IL-10) are enhanced by STAT3 activation (Weichhart et al., 2008). Thus, the TLR4 signaling can regulate the inflammatory response by modulating different transcriptions factors.

Obesity leads to an increase in IKK-β–NF-κB signaling, a primary regulator of inflammatory response, in the liver. This phenomenon is related to fatty liver accumulation, which activates IKK-β–NF-κB, resulting in pro-inflammatory cytokines and insulin resistance (Cai et al., 2005). In addition, myeloid cells IKK-β absence improves systemic insulin sensitivity (Arkan et al., 2005). Hypothalamic neurons IKK-β–NF-κB axis is also involved in obesity and insulin resistance (Zhang et al., 2008). This pathway is a target to non-acetylated salicylates drugs, which can emerge as a new treatment to glucose reduction in diabetic patients (Rumore and Kim, 2010). Furthermore, the IKKε deficiency protects from obesity, inflammation and insulin resistance (Chiang et al., 2009; Olefsky, 2009).

Other signaling protein is related to obesity (Hirosumi et al., 2002) and insulin resistance is cJun NH2-terminal kinase (JNK; Nguyen et al., 2005), a stress-responsive MAPK. Han et al. (2013) demonstrated that high-fat diet fed mice with JNK-deficient macrophages remains insulin-sensitive. However, these animals still develop obesity. On the other hand, Solinas et al. (2007) showed that JNK absence in non-hematopoietic cells reduces fat gain, possibly by increasing metabolic rate, besides insulin sensitivity improvement. JNK is also important to obesity induced-inflammation, since its deletion reduces M1 macrophage polarization, adipose tissue infiltration by macrophages and inflammatory cytokines levels (Solinas et al., 2007; Han et al., 2013). Between these cytokines, IL-6 is implicated to insulin resistance. Perry et al. (2015) demonstrated that macrophage IL-6 production via JNK pathway promotes lipolysis in white adipose tissue, which in turn are related to hepatic glucose increase production. Besides JNK peripheral role in obesity, studies provide evidences that JNK deficiency in the central nervous system, mostly of hypothalamic–pituitary axis, improves insulin sensitivity and reduces body mass (Belgardt et al., 2010; Sabio et al., 2010).

Between signaling proteins involved in inflammation and obesity, PI3K has emerged as an obesity treatment target (Wymann and Solinas, 2013; Perino et al., 2014). This class of enzymes catalyze the phosphorylation of inositol phospholipids to generate molecular messengers (Hawkins and Stephens, 2015). PI3Kβ and PI3Kγ isoforms inhibition are implicated in fat mass reduction by promoting increased energy expenditure in mice (Perino et al., 2014). Additionally, blockade of PI3Kγ reduces pro-inflammatory macrophages infiltration into adipose tissue (Kobayashi et al., 2011). In fact, different receptors stimulation (e.g., G protein-coupled or tyrosine kinases receptors) induces PI3Kγ activation, which promotes integrin α4β1 activation in myeloid cells, a fundamental step in cell migration (Schmid et al., 2011).

**Figure 1:**
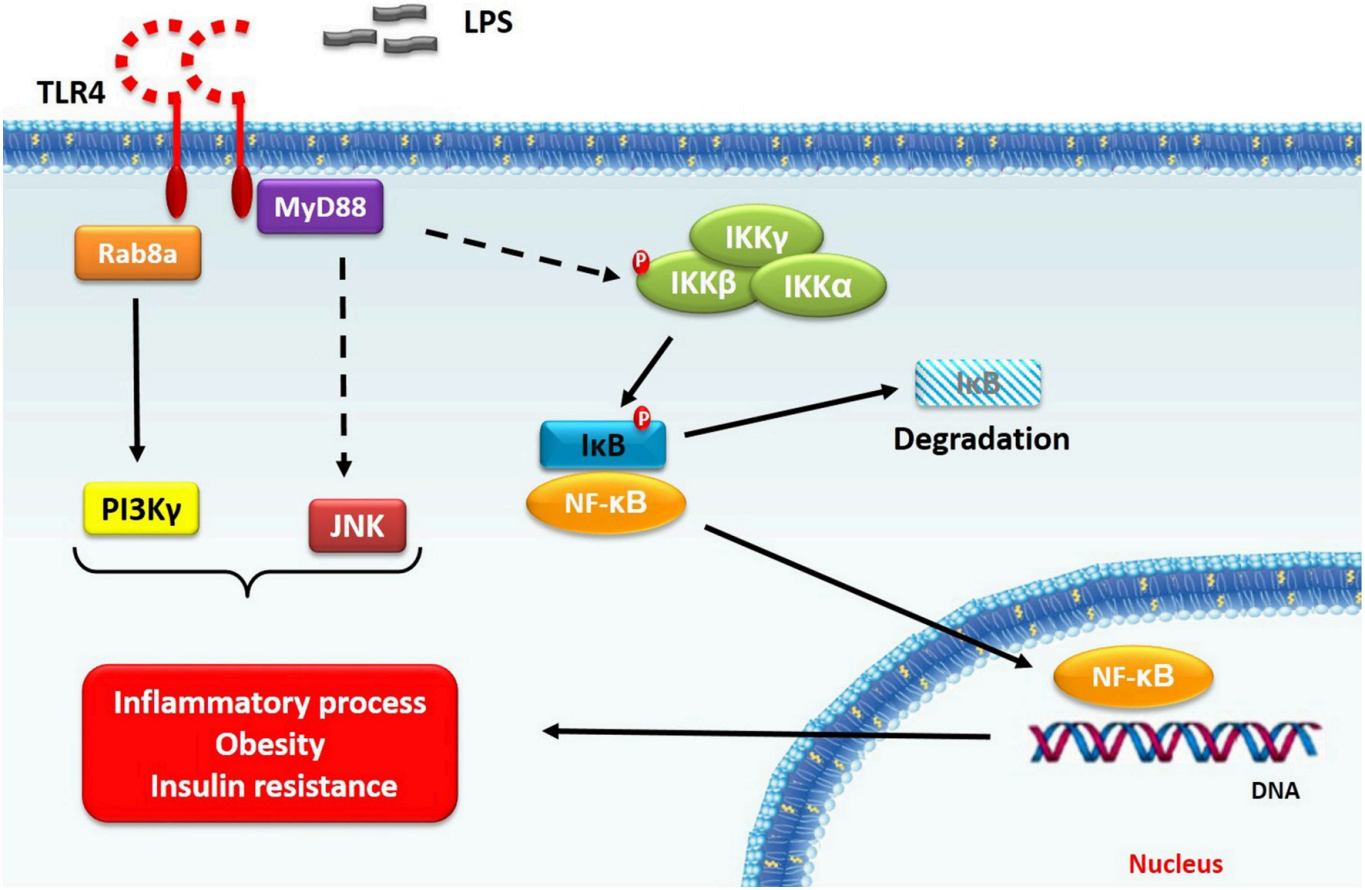
**TLR4 signaling in obesity**. TLR4 activation (i.e., after LPS stimulus) leads to signal transduction, which involves IKK-β–NF-κB classical pathway. After stimulation, MyD88 is recruited to TLR4 receptor to mediate downstream signaling, including IKK-β phosphorylation. Once activated, IKK-β phosphorylates IκB protein, which, in turn, release NF-κB complex. Besides this pathway, TLR4 signaling also results in PI3Kγ and JNK activation. Taken together, these signaling proteins play a fundamental role in inflammation, obesity and insulin resistance relationship. Note: dashed arrows indicate that other signaling intermediates are required.

Furthermore, PI3Kγ inhibition is also related to ameliorate obesity complications, mostly improving systemic insulin sensitivity (Becattini et al., 2011; Kobayashi et al., 2011). In this regard, TLR4/PI3Kγ axis is important not only for immune cells, but also for non-immune cells. Hepatocytes TLR4 absence, but not in myeloid cells, improved glucose tolerance and enhanced insulin sensitivity. Besides that, it also attenuates inflammatory response (Jia et al., 2014). Becattini et al. (2011) showed that PI3Kγ activity within non-hematopoietic cells promotes insulin resistance in high-fat diet fed mice. However, the relationship between TLR4 and cell signaling proteins (summarized in Figure 1), obesity and metabolic syndrome is not completely established.

## Conclusions and perspectives

**Figure 2:**
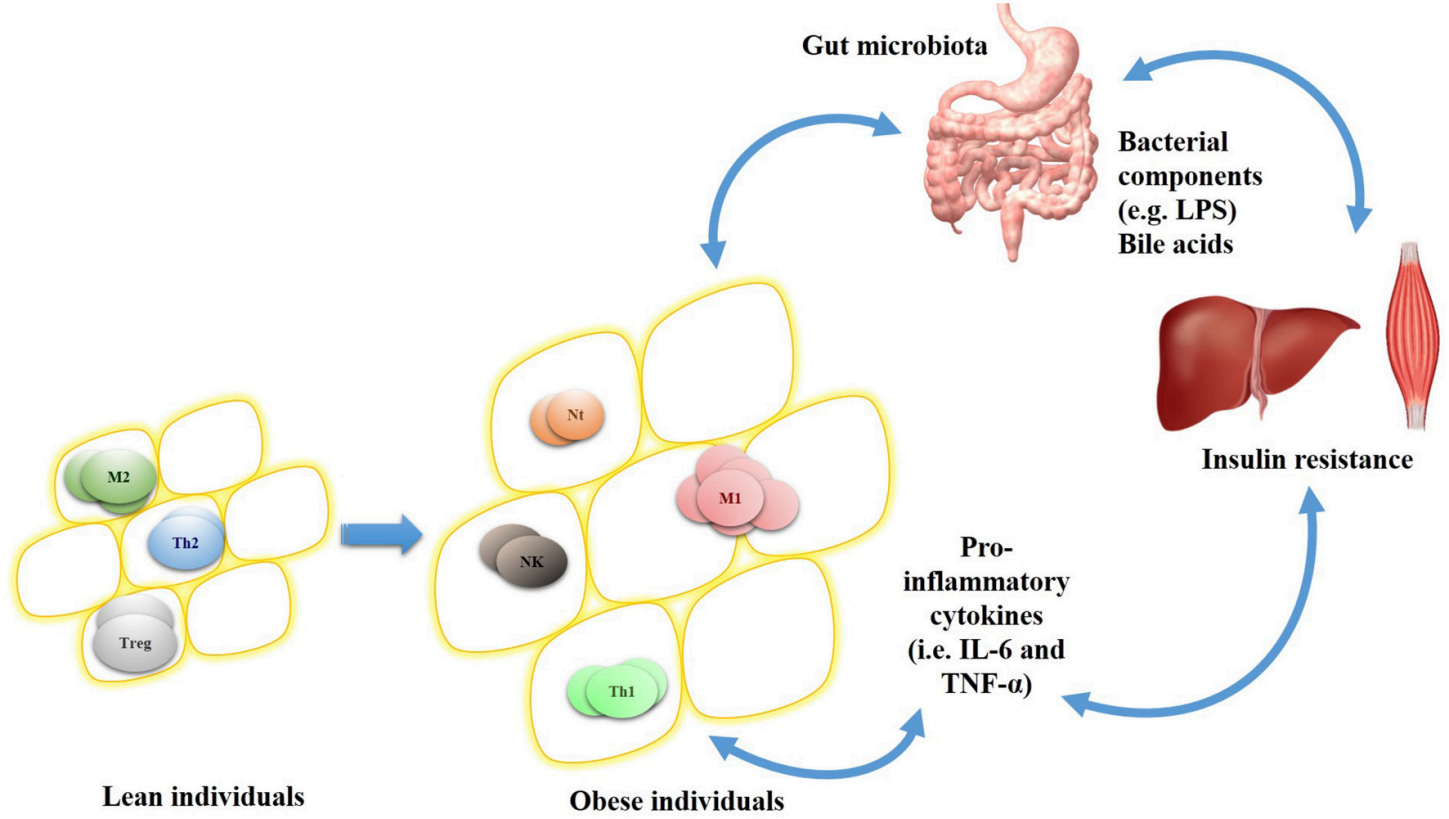
**Adipocytes-infiltrating immune cell profile in lean and obese individuals and relationship between gut microbiota, insulin sensitive organs, and inflammation**. In lean individuals, adipocytes cells (yellow circles) are infiltrated by anti-inflammatory cells (e.g., M2 macrophage and regulatory T cell [Treg]) and helper T lymphocyte 2 (Th2). On the other hand, obese individuals have hypertrophied adipocytes associated with pro-inflammatory cells (e.g., M1 macrophage and neutrophils [Nt]), NK and Th1 lymphocyte, which altogether induces pro-inflammatory mediators release. This inflammatory cell infiltration is influenced by the cytokines produced locally and also by host-gut microbiota interactions (e.g., bile acids and LPS influence), which in turn are directly associated to obesity and its complications (i.e., insulin resistance).

Although several pathophysiological studies of metabolic syndrome and obesity were reported, little has been done about translational research in this field. In this regard, gut microbiota emerges with a key role in these disorders by interacting with host metabolism (i.e., bile acid biotransformation) or by promoting immune responses (i.e., TLR activation and cytokines production). Hence, gut microbiota-driven inflammation may promote the activation of the signal transducers IKKβ, JNK, and PI3Kγ which in turn control obesity development, adipose tissue inflammation and insulin resistance. Further studies may consider the relationship between gut microbiota, immune system and obesity (Figure 2) as a novel scope for disorders prevention and health maintenance. This comprehension will allow the development of new specific targets and integrated strategies to modulate gut microbiota in order to improve or even treat metabolic syndrome and obesity.

### Conflict of interest statement

The authors declare that the research was conducted in the absence of any commercial or financial relationships that could be construed as a potential conflict of interest.
